# Gut Flora: Novel Therapeutic Target of Chinese Medicine for the Treatment of Cardiovascular Diseases

**DOI:** 10.1155/2019/3719596

**Published:** 2019-08-21

**Authors:** Yangwen Ou, Cuiping Zhang, Miaoen Yao, Lei Wang

**Affiliations:** ^1^Department of Cardiovascular Medicine, Second Affiliated Hospital of Guangzhou University of Chinese Medicine, Guangzhou 510120, China; ^2^Second Clinical College of Guangzhou University of Chinese Medicine, Guangzhou 510405, China

## Abstract

Cardiovascular disease (CVD) is one of the three major threats to human health identified by WHO. Dyslipidemia, hypertension, diabetes, and obesity are well established as common CVD risk factors. However, controversies exist on the effects of gut flora on cardiovascular disease (CVD). Current evidence suggests that gut microbiota is a double-edged sword for CVD risk, and its effects are largely determined by the metabolites of the gut microbiota. Trimethylamine N-oxide (TMAO), as one of the metabolites of gut flora, is consistently associated with higher CVD risk. A few studies have emerged providing early evidence about the safety and efficacy of traditional Chinese medicine (TCM) in treating cardiovascular diseases by regulating gut flora. In this article, we review and interpret the existing evidence as well as explore the potential of intestinal flora as novel therapeutic targets of traditional Chinese medicine for the prevention of cardiovascular disease (CVD).

## 1. Background

Cardiovascular disease (CVD) is a disease with high mortality. Either in China or America, CVD remains the primary cause of death and disability. CVD is responsible for approximately 33% of deaths in America and 40% of deaths in China [1, 2]. To our knowledge, hypertension, dyslipidemia, obesity, and diabetes have a serious impact on the occurrence and development of CVD. However, with the development of new-generation ribonucleic acid sequencing technology and metagenome technology, more and more studies have shown that gut flora is associated with CVD, hypertension, hyperlipidemia, and other diseases [3–7]. It is expected that regulating gut flora will become a new treatment for prevention of CVD.

Traditional Chinese medicine (TCM) has been used to treat CVD for thousands of years. Recent studies have found that traditional Chinese medicine can exert its therapeutic effect on CVD by regulating gut flora [8–10]. This prompts that gut flora may become a new therapeutic target for intervention of CVD.

## 2. Role of Gut Flora in CVD

There are more than 1,000 species of bacteria in the human gastrointestinal tract. Not only that, the total number of bacteria is 10^14^. The total number of genes is more than 100 times that of the human genome [11]. The gut flora is considered as the body's “second gene bank.” A few studies have demonstrated that the gut flora is associated with CVD [4]. With the development of microbial sequencing methods, metagenomic research, and bioinformatics, the study of gut flora and its association with cardiovascular diseases have been significantly improved.

### 2.1. Coronary Heart Disease

Studies have shown that obesity, inflammation, hyperlipidemia, and insulin resistance are the main risk factors for atherosclerotic heart disease. Intestinal metabolites produced by imbalance of intestinal flora play an important role in these risk factors [12, 13]. Short-chain fatty acids (SCFAs) are small molecular compounds, including butyrate, acetate, and propionate, produced by anaerobic bacteria digestion of food in the intestine. Butyrate can not only reduce the production of inflammatory factors, but also reduce the production of cell adhesion molecules to reduce the adhesion between macrophages and vascular endothelial cells [12, 14].

Karlsson et al. found that the number of *Roseburia* and *Eubacterium* producing butyrate was significantly reduced in atherosclerotic mice and patients with atherosclerotic symptoms [15]. TMAO is derived from lipid phosphatidylcholine. Intestinal bacteria convert the intake of lipid phosphatidylcholine into choline and trimethylamine (TMA). TMA is converted into trimethylamine nitrogen oxides (TMAO) catalyzed by flavin monooxygenase (FMO) in human liver [16]. It is appreciated that feeding choline-rich or TMAO-rich foods to mice accelerates the formation of atherosclerosis and alters cholesterol metabolism in the animal experiment [16]. However, when antibiotics or sterile mice were fed the same diet, the results showed that both TMAO produced by choline metabolism in food and the acceleration of atherosclerosis disappeared [16]. This inferred that TMAO could accelerate the occurrence of atherosclerosis.

In 2013, a large sample size study (*n* = 4007) of Tang et al. showed that elevated serum TMAO levels were associated with malignant cardiovascular events. The incidence of malignant cardiac events was 2.5 times higher in patients with a higher serum TMAO content than in patients with a lower serum TMAO content. Moreover, the risk ratio of TMAO was significantly higher than that of LDL. After adjusting for the traditional influencing factors and renal function, the elevated TMAO level was still an independent predictor of malignant cardiac events [17]. Several studies have also demonstrated that in patients with coronary syndrome, TMAO levels were associated with coronary plaque and the risk of cardiovascular events [18, 19]. In another cohort study, 935 PAD patients underwent selective angiography for cardiac evaluation, which found that increased TMAO levels were associated with a 2.7-fold increased risk of death. The highest TMAO quartile levels were predictive of 5-year mortality after adjusting for known risk factors, including inflammatory biomarkers and history of coronary artery disease [20].

Lipopolysaccharide (LPS), also known as endotoxin, is a component of the Gram-negative bacteria's cell membrane. LPS can be detected in atherosclerotic plaques. LPS promotes the expression of MMP9 by binding to Toll receptor. Matrix metalloproteinases then hydrolyze the extracellular matrix of the plaque cap, leading to plaque rupture, platelet aggregation, and arterial thrombosis. Ultimately it leads to cardiovascular events [21].

### 2.2. Heart Failure

The analysis of gut flora in patients with heart failure by 16S rRNA sequencing showed that the degree of heart failure was positively correlated with gut flora imbalance. This indicates that the imbalance of gut flora promotes the occurrence and development of heart failure [6].

There is a close relationship between TMAO and increased risk for future cardiovascular events. A recent study that enrolled 720 patients with stable heart failure (HF) showed that TMAO was associated with B-type natriuretic peptide (BNP). Adjusted for known risk factors (BNP and renal function), elevated plasma TMAO levels still predicted 5-year mortality risk [22]. Animal studies have also shown that dietary TMAO or choline exacerbates cardiac enlargement, left ventricular (LV) dysfunction, myocardial fibrosis, and pulmonary edema after transverse aortic constriction (TAC) in mice. This demonstrates a relationship between TMAO levels and heart failure susceptibility [23].

### 2.3. Hypertension

Hypertension is a common cardiovascular disease which is the result of the interaction of genetic factors and environmental factors. The regulation of blood pressure includes renin-angiotensin system, angiokinetic-prostaglandin system, and endothelial regulatory factors. In addition, neuroregulation and humoral regulation of blood vessels are also included. Among them, RAS is the main regulatory pathway. Angiotensin-converting enzyme (ACE) can catalyze the conversion of angiotensin 1 to angiotensin 2 and inactivate kallikrein. Probiotics can hydrolyze casein and milk protein to produce peptides that inhibit ACE [24, 25]. Pluznick found that SCFAs affect blood pressure through Olfr78 and Gpr41 receptors. SCFAs can regulate renin level through Olfr78 receptor and then affect peripheral vascular tension [26]. H_2_S is a kind of metabolite of intestinal flora. It was found that H_2_S could significantly reduce the mean arterial pressure in rats, especially in hypertensive rats. The effect of intracolonic administration was similar to that of intravenous administration, and the duration of intracolonic administration was longer. The vasodilation mediated by K-atp channel contributes to the antihypertensive effect of H_2_S [27].

This suggests that intestinal flora plays an important role in the regulation of blood pressure and may become a new target for antihypertensive therapy in the future.

### 2.4. Obesity and Type 2 Diabetes

The prevalence of obesity and type 2 diabetes is affected by environmental factors such as diets rich in fats or a sedentary living style. Studies have shown that obesity is associated with changes in gut flora. Studies have shown that obese patients have more Firmicutes and less Bacteroides in their guts [7]. Turnbaugh et al. found that sterile mice became obese by transplanting fecal bacteria from obese mice into the intestines of sterile mice [28]. Bacterial lipopolysaccharide (LPS), a potent endotoxin produced in the gut from Gram-negative bacteria, aggravates insulin resistance and obesity by triggering the secretion of inflammatory factors through the mCD14 and TLR4 signaling pathway. High-fat diets increased the amount of bacterial LPS in the gut [29]. Everard et al. discovered that *Akkermansia muciniphila*, a mucin-degrading bacterium, plays a critical role in obesity and its associated metabolic disorders, by increasing the fat mass, adipose tissue inflammation, and insulin resistance. Overweight and obese individuals had decreased levels of *A. muciniphila* which could be corrected using oligofructose prebiotics. Importantly, these effects were only observed when living *A. muciniphila* were administered, but not when heat-killed *A. muciniphila* was administered [30]. Bile acids are derivatives of cholesterol. Primary bile acids are synthesized in the liver. Primary bile acids are transformed into secondary bile acids under the action of gut flora [31]. Bile acids promote the absorption of postprandial nutrients. The farnesoid X receptor (FXR) and the G protein-coupled receptor (TGR5) are bile acid-activated nuclear receptors. Studies reveal that circulating bile acids activate the G protein-coupled receptor (TGR5) to promote GLP-1 secretion and reduce weight and increase insulin sensitivity [32, 33]. It was shown that total serum cholesterol in apolipoprotein E^−/−^ mice dropped 35% by inhibiting the apical sodium-codependent bile acid transporter [34]. Cariou found that FXR activation enhanced insulin signal transduction and insulin-stimulated glucose uptake [35].

### 2.5. Dyslipidemia

Hyperlipidemia is an important risk factor for coronary heart disease, which will accelerate the occurrence of atherosclerosis. Researchers from Kumamoto University analyzed the effects of gut flora changes on lipid metabolism; they have identified for the first time the molecular mechanism by which secondary bile acids produced by bacteria affect the concentration of blood fats [3]. Grill et al. reported that when fed with the same cholesterol feed, the plasma cholesterol content of aseptic animals was twice as high as that of bacterial animals. This suggests that gut flora can interfere with cholesterol absorption [36].

### 2.6. Dispute

At present, the effect of TMAO in the human body is controversial. TMAO can enhance protein folding and ligand binding and resist urea interference (e.g., in the mammalian kidney) [37]. Ma et al. found that TMAO plays a role in stabilizing proteins by increasing the amplitude of static components and weakening the strength of H-bonding between protein polar groups and water [38]. Collins et al. found that TMAO could delay the development of atherosclerosis in mice and protect blood vessels [39].

## 3. Mechanism of Gut Flora in the Etiology of TCM

“*Yellow Canons Internal Medicine*,” the classic work of TCM, holds that spleen and stomach are the acquired foundation. The physiological function of the spleen and stomach is that the spleen governs transportation and the stomach governs intake. After the diet entering the stomach of the human body, it is digested by the stomach and transported by the spleen, and the grains are transformed into qi, blood, and fluid needed by the human body. Finally, they are transmitted to all over the body to nourish organs and tissues. There are many species of bacteria in human intestinal microecosystem, such as Bifidobacterium, Bacteroides, Lactobacillus, , *Streptococcus faecalis*, and veronococcus. Enzymes produced by normal bacteria are involved in the digestion and absorption of nutrients (carbohydrates, proteins, and lipids). This is very similar to the physiological function of spleen and stomach in TCM.

However, under the stress of various pathogenic factors, including improper diet and imbalance between work and rest, the spleen and stomach will be damaged. Consequently, the subtle substance of the grain will be transformed into “phlegm and turbidity” due to the dysfunction of spleen and stomach. Traditional Chinese medicine believes that many diseases are caused by phlegm. Animal experiments showed that mulberry leaf da huang decoction reduced the intestinal flora diversity index of rats with spleen deficiency syndrome, such as Lactobacillus and Bifidobacterium, to different degrees [40]. Jia et al. found that ERIC-PCR fingerprint of intestinal flora of rats with spleen deficiency syndrome changed significantly compared with those before modeling, and their diversity index decreased [41]. This indicates that senna leaf and rhubarb can disrupt intestinal microecological balance and lead to microflora imbalance. In other words, qi deficiency syndrome directly affects the balance of intestinal flora.

The theory of TCM holds that the pathogenesis of CVD is characterized by imbalance of yin and yang, phlegm, and blood stasis. The results showed that endogenous phlegm dampness was caused by stress factors such as deficiency of spleen and stomach, emotional disorder, and qi deficiency caused by improper diet. The excessive phlegm and dampness were absorbed into meridian and result in the blood stasis. Intermingled phlegm and blood stasis obstruct the heart meridian, leading to the occurrence of chest pain. At present, the view that “phlegm is related to blood stasis” has become the common pathological mechanism for cardiovascular and cerebrovascular diseases.

**Figure 1:**
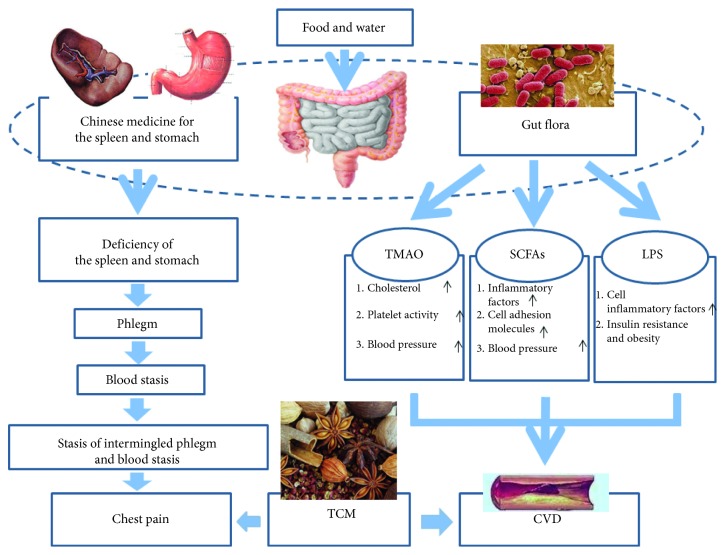
Gut flora and possible molecular pathways linked to TCM and cardiovascular diseases. CVD, cardiovascular diseases; LPS, lipopolysaccharide; SCFAs, short-chain fatty acids; TCM, traditional Chinese medicine; TMAO, trimethylamine N-oxide.

Abnormal transportation and transformation of spleen-stomach are closely related to gut flora disorder. Gut flora converts phosphatidylcholine and L-carnitine contained in food into TMA. It is a biomarker of “phlegm and turbidity.” Subsequently, TMA is converted into TMAO in the liver after it enters the blood [16]. Wang et al. found that increased levels of TMAO not only impair the reverse transport of cholesterol and its catabolism, but also increase platelet activity and promote thrombosis [16]. This is the pathological basis of blood vessel occlusion described as “stasis of intermingled phlegm and blood stasis.” (Figure 1).

## 4. Therapeutic Intervention with TCM

TCM decoction taken orally is the main route of administration. The herbal remedy comes in contact directly with the gut flora during the metabolic process. The herb will then be transformed into active ingredients by bacterial enzymes in the intestine. Several studies have shown that TCM monomers or compounds might improve the symptoms of cardiovascular diseases by altering the gut flora, and hence reminds us of the important role of the gut flora in herbal metabolism (Table 1).

### 4.1. Berberine

Berberine (BBR) is an isoquinoline alkaloid extracted from *Rhizoma coptidis* plants. Kong et al. demonstrated that BBR reduces serum cholesterol levels by increasing the expression of low-density lipoprotein receptor (LDLR) in the liver [36]. Furthermore, BBR has recently been demonstrated to significantly reduce fasting and postprandial blood sugar in patients [42]. Animal studies have shown that metformin and BBR have similar effects on high-fat diet-induced structural changes induced by gut flora. They can reduce insulin resistance by increasing the abundance of bacteria produced by short-chain fatty acids and reducing endotoxemia [8, 9].

### 4.2. *Ganoderma lucidum*

*Ganoderma lucidum* is a type of medicinal mushroom. Chang et al. demonstrated that *Ganoderma lucidum* mycelium (WEGL) could reduce obesity, chronic inflammation, and insulin resistance [43]. Its pharmacological effect is achieved by regulating gut flora. WEGL can reverse gut flora disorders by reducing the ratio of Firmicutes to Bacteroidetes and the number of *E. coli*, while increasing levels of several beneficial bacteria, such as *Clostridium* and *Eubacterium*. In addition, it can reduce metabolic endotoxemia and maintain the integrity of the intestinal barrier. This is significant for the prevention and treatment of coronary heart diseases.

### 4.3. Ginsenosides

Ginseng is a traditional Chinese medicine, which has the function of reinforcing vital energy, reviving yang for resuscitation. Ginsenoside has potentially beneficial effects on CVD, including antioxidation, reducing platelet adhesion, and improving lipid metabolism. Ginsenoside Rb1 has a protective effect on vascular endothelial cells damaged by oxidative stress or oxidized low-density lipoprotein. It protects endothelial cells in many ways, such as increasing the expression of eNOS, antioxidant enzyme activity, protecting mitochondria, and upregulating the expression of vascular endothelial growth factor [44, 45]. Ginsenoside Rg1 and Ginsenoside Re have significant angiopoietin-like effects. Liang et al. treated myocardial infarction rats with G-Rg1 and then found that G-Rg1 could promote tissue regeneration and prevent the expansion of the infarction area [46].

### 4.4. Gegen Qinlian Decoction (GQD)

Recent studies have discovered that GQD, a Chinese herbal formula, can alleviate type 2 diabetes (T2D). In a double-blind, randomized controlled study of 187 T2D patients, patients were randomized to receive high-dose (HD), medium-dose (MD), and low-dose (LD) GQD or placebo for 12 weeks. GQD could significantly reduce the levels of fasting blood glucose (FBG) and glycated hemoglobin (HbA1c), but also apparently increase the levels of HOMA-*β*. Plasma levels of tumor necrosis factor A, adiponectin, and serum amyloid A were not significantly different in the four groups. GQD apparently increased the level of *Faecalibacterium prausnitzii*, a beneficial bacterium that is negatively associated with FBG, HbA1c, and 2-h postprandial blood glucose levels. These suggest that GQD may play a role in the treatment of T2D by regulating gut flora [10].

## 5. Conclusion and Future Perspective

Increasing evidence has shown that the gut flora plays an important role in the occurrence and development of many diseases. Regulation of intestinal bacterial metabolism may be a new pharmacological target for the treatment of CVD. This requires a more systematic understanding of how intestinal microorganisms convert diets or traditional Chinese medicines into metabolites that interact with surrounding tissues and organs. High-throughput analysis and bioinformatics can help us to study the changes and regularities of endogenous global metabolism. Furthermore, it is conducive to understand the mechanism of how metabolites interact with host receptors. Traditional Chinese medicine can treat diseases by regulating intestinal flora to change the metabolism of the body. Therefore, the identification of gut microbiota and their metabolites will be significant in developing individualized intervention strategies.

## Figures and Tables

**Table 1:** Summary of the efficiency of Chinese medicine.

Diseases	Type 2 diabetes and obesity	Obesity	Type 2 diabetes	CVD
Herbs/decoction	Berberine [8, 9, 42]	*Ganoderma lucidum* [43]	Gegen Qinlian decoction [10]	Ginsenosides [44–46]
Source/component	*Rhizoma coptidis* plants	*Ganoderma lucidum*	Gegen (Radix Puerariae), Huang Qin (Radix Scutellariae), Huang Lian (*Rhizoma coptidis*), Gancao (Honey-fried Licorice Root)	Ginseng
Species	Humans; rat	Mouse	Human	Cell; rat
Alteration of intestinal flora	SCFA-producing bacteria ↑ (Allobaculum, Bacteroides, Blautia, Butyricicoccus, and Phascolarctobacterium)	The ratio of Firmicutes to Bacteroidetes ↓; the number of *Escherichia coli* ↓; levels of several beneficial bacteria ↑(*Clostridium*, *Eubacterium*)	Level of *Faecalibacterium prausnitzii*↑	None
Therapeutical effect	Fasting and postprandial blood sugar ↓; insulin resistance ↓; inflammation ↓	Chronic inflammation ↓; insulin resistance ↓; metabolic endotoxemia ↓; maintain the integrity of the intestinal barrier	Levels of FBG and HbA1c ↓; levels of HOMA-*β* ↑	Expression of eNOS and vascular endothelial growth factor ↓; tissue regeneration ↑; expansion of infarction area ↓

eNOS, endothelial nitric oxide synthase; FBG, fasting blood glucose; HbA1c, glycated hemoglobin; HOMA-*β*, homeostasis model assessment *β*; SCFA, short-chain fatty acid.↑: increase or promote; ↓: reduce or alleviate.
